# Reliability of maternal recall of delivery and immediate newborn care indicators in Sarlahi, Nepal

**DOI:** 10.1186/s12884-021-03547-5

**Published:** 2021-01-25

**Authors:** Emily D. Carter, Karen T. Chang, Luke C. Mullany, Subarna K. Khatry, Steven C. LeClerq, Melinda K. Munos, Joanne Katz

**Affiliations:** 1 Department of International Health, Johns Hopkins Bloomberg School of Public Health, 615 North Wolfe Street, Baltimore, MD USA; 2 Division of Reproductive Health, CDC National Center for Chronic Disease Prevention and Health Promotion, Atlanta, GA USA; 3 Johns Hopkins University Applied Physics Laboratory, Laurel, MD USA; 4 Nepal Nutrition Intervention Project–Sarlahi, Lalitpur, Nepal

**Keywords:** Birth, Intrapartum, Recall, Reliability, Survey

## Abstract

**Background:**

The intrapartum period is a time of high mortality risk for newborns and mothers. Numerous interventions exist to minimize risk during this period. Data on intervention coverage are needed for health system improvement. Maternal report of intrapartum interventions through surveys is the primary source of coverage data, but they may be invalid or unreliable.

**Methods:**

We assessed the reliability of maternal report of delivery and immediate newborn care for a sample of home and health facility births in Sarlahi, Nepal. Mothers were visited as soon as possible following delivery (< 72 h) and asked to report circumstances of labor and delivery. A subset was revisited 1–24 months after delivery and asked to recall interventions received using standard household survey questions. We assessed the reliability of each indicator by comparing what mothers reported immediately after delivery against what they reported at the follow-up survey. We assessed potential variation in reliability of maternal report by characteristics of the mother, birth event, or intervention prevalence.

**Results:**

One thousand five hundred two mother/child pairs were included in the reliability study, with approximately half of births occurring at home. A higher proportion of women who delivered in facilities reported “don’t know” when asked to recall specific interventions both initially and at follow-up. Most indicators had high observed percent agreement, but kappa values were below 0.4, indicating agreement was primarily due to chance. Only “received any injection during delivery” demonstrated high reliability among all births (kappa: 0.737). The reliability of maternal report was typically lower among women who delivered at a facility. There was no difference in reliability based on time since birth of the follow-up interview. We observed over-reporting of interventions at follow-up that were more common in the population and under-reporting of less common interventions.

**Conclusions:**

This study reinforces previous findings that mothers are unable to report reliably on many interventions within the peripartum period. Household surveys which rely on maternal report, therefore, may not be an appropriate method for collecting data on coverage of many interventions during the peripartum period. This is particularly true among facility births, where many interventions may occur without the mother’s full knowledge.

**Supplementary Information:**

The online version contains supplementary material available at 10.1186/s12884-021-03547-5.

## Background

The intrapartum and immediate postpartum periods are a time of high mortality risk for newborns and mothers. Neonatal deaths account for almost half of all under-five deaths, and mortality within this period has been difficult to reduce [1]. Numerous interventions exist to minimize risk to both newborns and mothers in the peripartum period. Data on population coverage of these interventions are needed for health programs to ensure that interventions are reaching those in need. However, such data are often scarce or unreliable. Information on the content or quality of care administered during labor and delivery at health facilities is frequently un- or under-documented, and rarely reported in national health information systems. Even less information is available for deliveries occurring outside of the government health system. Population-based surveys of women, with questions focused on care received during recent deliveries, are often used to capture data on intervention coverage for both facility and non-facility deliveries. However, an increasing number of studies have shown that women are often unable to accurately report on the content of care received, particularly for interventions occurring during the peripartum period [2–6]. A qualitative study by Yoder and colleagues in Bangladesh and Malawi found mothers had difficulty understanding some terminology related to peripartum care and comprehending questions about the timing of events following birth [7]. Work by McCarthy and colleagues suggests that pain, fatigue, and relief of a successful delivery may distract women from noting the care they received [5]. Further, women may not be told about the care they receive, such as the type of injection, or maybe more or less likely to report an intervention due to social desirability biases, sometimes providing responses that are believed to be viewed more positively by others. To date, validation studies of peripartum care have focused on women delivering in health facilities and have primarily been conducted in sub-Saharan Africa or Latin America.

We assessed the reliability of maternal report of delivery care and immediate newborn care for a sample of both home and health facility births in Nepal. Within the first 3 days after delivery, mothers were asked to report on interventions received during the peripartum period. These same women were visited between 1 and 24 months later and asked to recall the interventions they received during the peripartum period. Maternal report at both time points was compared to assess the reliability of maternal recall of peripartum health interventions. We also assessed potential variation in recall reliability by characteristics of the birth, the mother, and intervention prevalence.

## Methods

### Study setting

The study was conducted in the Sarlahi district of Nepal, bordering the Indian state of Bihar, to the south. Residents are primarily Hindu and agrarian. In the study area, approximately half of births occur at home and half in health facilities.

### Parent trial

Data on interventions received during the peripartum period were collected through a parent trial conducted jointly by the Nepal Nutrition Intervention Project – Sarlahi (NNIPS) and our local partner organization, Nepal Netra Jyoti Sangh under the auspices of the Social Welfare Council of the Government of Nepal. Women and their newborns were enrolled in a randomized community-based trial to investigate the impact of full-body newborn massage with sunflower seed oil on newborn deaths and infections. The trial was registered at ClinicalTrials.gov (NCT01177111). The study took place in 34 Village Development Committees in the rural district of Sarlahi, Nepal, between November 2010 and January 2017.

Pregnant women were identified in the community and followed through delivery. Pregnant women participating in the trial were given clean birth kits, chlorhexidine (CHX) for application to the cut umbilical stump, deworming tablets, and counseling on early breastfeeding, thermal care, umbilical cord care, delivery care, postnatal care, and danger signs during labor and postnatal period. Mothers were visited as soon as possible following delivery, typically within 24 h, and asked to report on the date/time of delivery, circumstances of labor and delivery, the health status of the mother and newborn, and baby’s weight. The wording of relevant delivery and immediate postpartum intervention questions administered to the mother at the first visit are listed in Supplementary Questionnaire 1. Additional interviews conducted throughout the first month (days 3, 7, 10, 14, 21, and 28) focused on maternal report and directly observed aspects of newborn health.

### Reliability substudy

**Fig. 1:**
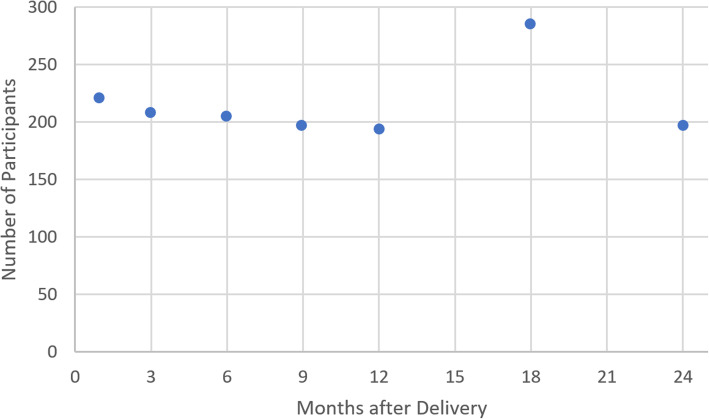
Number of study participants by number of months since delivery

We randomly selected a subset of mother/child pairs that participated in the parent trial and revisited these women between April and September 2016. Each selected mother was visited at home and asked to report on interventions and events in the peripartum period, including labor and delivery, immediate newborn care, postnatal care, and illness and care within the first 7 days of life using standard questions from the Demographic and Health Survey (DHS) or Multiple Indicator Cluster Survey (MICS) where applicable (see Supplementary Questionnaire 2). Mothers who had a singleton live birth and who were visited at home within 72 h after delivery were eligible. We interviewed approximately equal numbers of mothers at each of seven follow-up time periods: 1, 3, 6, 9, 12, 18, or 24 months after birth (Fig. 1).

Selected mothers were requested to participate in the validation substudy conducted by study staff through an oral consent process in either the Nepali or Maithili language, both of which are spoken in the area. Those who consented to participate were asked to recall care during delivery and the immediate postnatal period prior to discharge.

### Ethical approval

The parent trial and validation substudy were approved by the Johns Hopkins Bloomberg School of Public Health Institutional Review Board in Baltimore, USA. In Nepal, approval was received from the Tribhuvan University Institute of Medicine, Kathmandu (parent trial) and the Nepal Health Research Council, Kathmandu (substudy).

### Data analysis

The reliability of each indicator was assessed by comparing what mothers reported immediately after delivery against what was reported at the follow-up survey. We assessed the reliability of 14 indicators, including the use of items within the clean birth kit, injections given during labor / delivery, immediate newborn care, cord care, and early initiation of breastfeeding. Indicators related to immediate newborn care were defined as practices generally occurring between the delivery of the child and the delivery of the placenta. Questions about the application of CHX or other substances to the cord stump were limited to applications immediately following delivery. Early breastfeeding initiation was defined as putting the child to the breast within the first hour after delivery.

For both the initial assessment and follow-up survey, we assessed the proportion of mothers who responded “don’t know” (DK) when asked whether the intervention or practice occurred. Each mother was asked to identify each injection given during labor and delivery. If the mother reported she could not identify a specific injection and did not state oxytocin or ergometrine was given their response on “injectable Oxytocin / Ergometrine given during delivery” was classified as “DK”. The same logic was used for classifying the use of chlorohexidine. Excluding DK responses, we calculated the observed percent agreement, expected percent agreement, and kappa of maternal recall for each indicator. A sensitivity analysis maintaining DK responses as a separate response category was also conducted. The kappa statistic (κ) was used to measure the test-retest reliability of maternal report after excluding agreement due to chance. Chance expected percent agreement (p_e_) was defined as classification at random assuming probability equal to the overall proportion of yes and no responses at the initial (time 1) and follow-up (time 2) interview. Observed percent agreement (p_o_) was calculated as the number of mothers reporting either receiving or not receiving a specific intervention at both the initial and follow-up survey. The kappa statistic was calculated as the difference in the expected and observed agreement over one minus the expected change agreement (Formula 1).

$$ {p}_e=\left({p}_{y{es}_{t1}}\ast {p}_{y{es}_{t2}}\right)+\left({p}_{no_{t1}}\ast {p}_{no_{t2}}\right) $$

$$ {p}_o={p}_{y{es}_{t1}}\ast \left({p}_{y{es}_{t2}\mid {yes}_{t1}}\right)+{p}_{n{o}_{t1}}\ast \left({p}_{n{o}_{t2}\mid {no}_{t1}}\right) $$

$$ \kappa =\frac{p_o-{p}_e}{1-{p}_e} $$

A κ = 1 is considered perfect agreement and κ = 0 is considered no agreement beyond that expected by chance alone. We interpreted κ values of greater than 0.4 as indicating moderate reliability and values greater than 0.6 as indicating strong reliability. We also calculated the proportion of women who changed their responses from 1) not receiving an intervention at the initial assessment but reported receipt during the follow-up survey (over-report), and 2) reported receiving an intervention at the initial assessment but did not report the intervention during the follow-up survey (under-report). Analyses were stratified by site of delivery, dichotomized as facility deliveries and home deliveries.

At the level of the individual respondent, we assessed potential variation in the reliability of maternal report by characteristics of the mother or birth event we hypothesized could potentially alter women’s ability to recall events around delivery. We used multivariable logistic regression to assess differences in percent agreement, over-reporting, and under-reporting of each indicator by time between birth and follow-up interview (continuous variable), child sex, maternal education (none versus any), maternal age, parity, birth location, and presence of delivery complication. Delivery complications were defined as maternal report of complications during delivery such as excessive bleeding, prolonged labor, convulsions, fever, or obstructed labor at the initial interview. We also looked for unadjusted differences in percent agreement by time since birth using binned categories for the best performing indicators.

We also assessed associations at the indicator level between the reliability of maternal recall and underlying intervention coverage or prevalence, based on initial maternal report. We calculated the unadjusted associations between intervention prevalence and indicator estimates of percent agreement, κ, the proportion of women over-reporting, and the proportion of women under-reporting each indicator. Where significant associations were identified, we calculated the proportion of variability in indicator reliability explained by differences in underlying intervention prevalence. All analyses were conducted in Stata version 14.0 (StataCorp, College Station, TX, USA).

## Results

Of the 1892 women selected for the reliability study, 363 were not available, 5 had moved permanently, 3 had died, 4 refused to participate, and 1517 were consented and interviewed (see Supplementary Figure 1). There was no significant difference in the characteristics of those women who were and were not available to participate. After excluding 15 participants (birth assessment > 72 h after birth [*n* = 3], twin delivery [*n* = 1], repeat participation due to multiple eligible births [*n* = 11]), 1502 mother/child pairs were included in the substudy. Of these, 220 were enrolled in the one-month recall group, 207 in the three-month group, 205 in the six-month group, 196 in the nine-month group, 193 in the 12-month group, 284 in the 18-month group, and 197 in the 24-month group (Fig. 1).

More than half of newborns were male (55.5%), and a majority of births occurred in the home (53.8%) (Table 1). The mean recall period was 10.8 months. The mean age of mothers was 23.9 years at the time of delivery. Most mothers had no schooling (68.3%) and had prior children (71.4%). Participants were nearly universally of Madhesi (people of the plains) ethnic origin (96.2%), frequently lacked a household latrine (71.2%), but owned some type of land (97.4%). The substudy sample was comparable to the parent trial sample, but the parent trial sample was more balanced by child sex (male = 51.3%). Stratifying by the site of delivery, women who delivered in health facilities were younger and more educated than women who delivered at home (see Supplementary Table 1). They also were more often having their first child (41.6% vs 17.3%) and more often reported birth complications (28.9% vs 8.7%) compared to women who delivered at home.

**Table 1:** Characteristics of sample

	n	Mean / %	95% CI
Recall period (weeks)	1502	47.0	(45.3, 48.8)
Child sex			
Male	834	55.5%	(53.0, 58.0)
Female	668	44.5%	(42.0, 47.0)
Place of Delivery			
Home	773	53.8%	(51.3, 56.3)
Facility	728	46.2%	(43.7, 48.7)
Birth complications			
None	1230	82.0%	(80.0, 83.9)
Reported	270	18.0%	(16.1, 20.0)
Parity			
Primiparous	430	28.6%	(26.3, 30.9)
Second	382	25.4%	(23.3, 27.7)
Third	311	20.7%	(18.7, 22.8)
Fourth +	380	25.3%	(23.2, 27.6)
Maternal Age (yrs) at delivery			
< 20	348	23.2%	(21.2, 25.4)
20–29	976	65.0%	(62.6, 67.4)
30–39	162	10.8%	(9.3, 12.5)
40+	14	0.9%	(0.6, 1.6)
Maternal education			
None	1025	68.3%	(65.9, 70.6)
Any	476	31.7%	(29.4, 34.1)
Ethnicity			
Madhesi	1444	96.2%	(95.1, 97.1)
Pahadi	57	3.8%	(2.9, 4.9)
HH electricity			
No electricity	293	19.5%	(17.6, 21.6)
Had electricity	1208	80.5%	(78.4, 82.4)
HH latrine status			
No latrine	1069	71.2%	(68.8, 73.4)
Had latrine	432	28.8%	(26.6, 31.2)
Land ownership			
Did not own land	39	2.6%	(1.9, 3.5)
Owns land	1462	97.4%	(96.5, 98.1)

As an initial assessment of intervention recall and question comprehension, we assessed the proportion of mothers that responded DK when asked about each intervention or practice at the initial post-birth assessment and at follow-up (Table 2). Only a handful of indicators (4 for facility births, 1 for home births) had a greater than 5% DK response rate during the initial assessment, but this increased to 9 and 5 indicators, respectively, at follow-up. Recall of the type of injection received the highest proportion of DK responses. During the initial interview, most mothers reported receiving multiple injections during delivery, and mothers could not identify approximately 90% of the injections they reportedly received. Recall of type of injection was also poor at the follow-up survey, however it was partially masked by a reduction in the number of injections that mothers reported receiving during delivery. During the initial assessment, women reported receiving 1.83 injections on average, which fell to 1.27 during the follow-up survey.

**Table 2:** Rate of “don’t know” responses by question, during initial and follow-up interview, by site of delivery

	Facility Births				Home Births			
	Initial		Follow-up		Initial		Follow-up	
	**n**	% DK	**n**	% DK	**n**	% DK	**n**	% DK
Clean Birth Kit - Any Item Used	691	2.6	693	3.8	808	0	808	0.4
Clean Birth Kit - Sheet Used	691	2.9	693	7.5	808	0.1	808	1.5
Clean Birth Kit - Soap Used	691	7.2	693	8.1	808	0.7	808	2.4
Any injection given during delivery	693	0	683	0	808	0	808	0.2
Type of injection given	1535	90.0	1016	86.2	1220	89.2	896	87.8
Newborn wrapped before placenta delivered	693	2.3	693	13.3	808	0.1	808	7.8
Newborn washed before placenta delivered	693	2.2	693	20.5	808	0	808	9.2
Newborn wiped with cloth before placenta delivered	693	3.3	693	19.6	808	0	808	12.5
Newborn placed on mother’s chest or arms before placenta delivered	693	1.7	693	3.3	808	0.1	808	3.5
Cord cut after placenta delivered	693	4.8	693	19.0	808	0.2	808	5.7
Cord cut with new blade	693	10.4	693	28.7	808	0.2	808	1.6
Anything applied to cord stump immediately after delivery	693	5.3	693	15.4	808	0.1	808	2.5
Type of substance applied to cord immediately after delivery	758	0.3	517	1.2	775	0	780	0.1
Breastfeeding initiated in first hour	693	0.1	693	0.1	808	0.1	808	1.0

The proportion of women who gave DK responses was higher overall among women that delivered at a facility, compared to women who delivered at home, at both the initial and follow-up interview. For example, at the initial assessment, 10.4% of women who delivered at a facility could not report whether a new blade had been used to cut the umbilical cord compared to just 0.2% of women who delivered at home. This was true for other cord care indicators, with 5–10% of facility-delivering mothers reporting DK at the initial assessment increasing to 15–29% reporting DK at the follow-up survey. Indicators involving the timing of wiping, wrapping, bathing, and cord-cutting had > 5% DK responses at follow-up across both home and facility births, but the proportion of DK responses was much higher for facility births.

The reported intervention coverage, percent agreement, and kappa values of each indicator are presented in Table 3, and for facility and home deliveries separately in Table 4. The majority of indicators had a high observed percent agreement, but most kappa values were below 0.4, indicating agreement was primarily due to chance. Among all observations, three indicators showed moderate reliability with kappa values greater than 0.4, including “any part of the clean birthing kit used for the delivery,” “sheet from clean birthing kit used for the delivery,” and “cord cut after placenta delivered.” Only one indicator, “received any injection during delivery,” demonstrated high reliability with a kappa of 0.737. Stratifying by place of delivery, the reliability of maternal report was much lower among women who delivered at a facility. Only 5 out of 14 indicators had greater than 70% agreement among facility deliveries, and none had kappa values above 0.4. Among home deliveries, 11 out of 14 indicators had > 70% agreement, and both use of a clean birthing kit and any injection during delivery had kappa values above 0.4 and 0.6 respectively. Inclusion of DK as a separate response category did not significantly alter the reliability of any indicator, with the exception of “received oxytocin / ergometrine during delivery,” where reliability improved due to the high number of DK responses (see Supplementary Tables 2 & 3).

**Table 3:** Reliability of maternal report of immediate newborn care indicators

	Proportion report receiving intervention				Observed Agreement			Expected Agreement	Kappa	K + > 0.4 ++ > 0.6
	Initial		Follow-up							
	n	%	n	%	n	%	95% CI	%		
Clean Birth Kit - Any Item Used	1482	71.1	1473	80.9	1456	82.6	(80.5–84.4)	63.7	0.520	+
Clean Birth Kit - Sheet Used	1482	63.2	1473	71.7	1456	75.1	(72.8–77.2)	56.1	0.432	+
Clean Birth Kit - Soap Used	1482	64.0	1473	69.5	1456	69.4	(67.0–71.8)	55.8	0.309	
Any injection given during delivery	1502	74.4	1500	68.3	1500	89.2	(87.5–90.7)	58.9	0.737	++
Injectable Oxytocin / Ergometrine given during delivery	439	98.6	523	98.8	371	99.7	(98.1–99.9)	99.7	0	
Newborn wrapped before placenta delivered	1485	50.6	1347	58.9	1335	55.7	(53.0–58.3)	50.1	0.112	
Newborn washed before placenta delivered	1487	0.2	1286	5.8	1276	94.2	(92.8–95.4)	94.1	0.022	
Newborn wiped with cloth before placenta delivered	1479	44.9	1265	58.8	1249	57.6	(54.8–60.3)	49.0	0.167	
Newborn placed on mother’s chest or arms before placenta delivered	1489	21.1	1451	15.6	1439	78.2	(76.0–80.2)	69.7	0.280	
Cord cut after placenta delivered	1467	64.6	1324	72.3	1301	80.9	(78.6–82.9)	57.9	0.545	+
Cord cut with new blade	1427	68.7	1290	91.7	1244	81.6	(79.3–83.6)	71.8	0.346	
Anything applied to cord immediately after delivery	1464	94.7	1375	92.8	1350	90.4	(88.7–91.8)	89.0	0.128	
CHX applied to cord stump immediately after delivery	1462	89.1	1368	91.9	1342	87.1	(85.2–88.8)	83.8	0.206	
Breastfeeding initiated in first hour	1500	35.4	1493	29.9	1491	64.7	(62.2–67.0)	55.8	0.201

**Table 4:** Reliability of maternal report of immediate newborn care indicators, by site of delivery

	Proportion report receiving intervention				Observed Agreement			Expected Agreement	Kappa	K + > 0.4 ++ > 0.6
	Initial		Follow-up							
	n	%	n	%	n	%	95% CI	%		
**Facility Delivery**										
Clean Birth Kit - Any Item Used	673	44.0	667	62.2	650	66.8	(63.1–70.3)	48.7	0.352	
Clean Birth Kit - Sheet Used	673	41.3	667	54.6	650	67.2	(63.5–70.7)	49.2	0.355	
Clean Birth Kit - Soap Used	673	37.7	667	57.1	650	62.9	(59.1–66.6)	48.2	0.284	
Any injection given during delivery	693	97.0	693	88.2	693	88.9	(86.3–91.0)	85.9	0.215	
Injectable Oxytocin / Ergometrine given during delivery	48	6.2	118	1.7	22	95.4	(70.4–99.5)	95.5	0	
Newborn wrapped before placenta delivered	677	62.5	601	71.0	590	60.0	(56.0–63.9)	55.4	0.102	
Newborn washed before placenta delivered	678	0.3	551	8.7	541	91.3	(88.6–93.4)	91.0	0.034	
Newborn wiped with cloth before placenta delivered	670	63.0	557	72.2	541	56.9	(52.7–61.0)	55.6	0.030	
Newborn placed on mother’s chest or arms before placenta delivered	681	43.3	670	27.0	659	60.7	(56.9–64.4)	52.9	0.166	
Cord cut after placenta delivered	660	22.3	561	39.6	538	59.3	(55.1–63.4)	55.6	0.082	
Cord cut with new blade	621	30.4	494	78.7	449	51.0	(46.4–55.6)	41.7	0.160	
Anything applied to cord immediately after delivery	656	93.9	586	86.7	561	84.0	(80.7–86.8)	83.1	0.049	
CHX applied to cord stump immediately after delivery	654	82.9	580	85.5	554	77.1	(73.4–80.4)	74.1	0.114	
Breastfeeding initiated in first hour	692	43.9	692	40.0	691	60.6	(56.9–64.2)	51.2	0.193	
**Home Delivery**										
Clean Birth Kit - Any Item Used	808	93.8	805	96.4	805	95.4	(93.7–96.7)	90.6	0.509	+
Clean Birth Kit - Sheet Used	808	81.6	805	85.8	805	81.5	(78.7–84.0)	72.6	0.325	
Clean Birth Kit - Soap Used	808	85.9	805	79.6	805	74.8	(71.7–77.7)	71.3	0.121	
Any injection given during delivery	808	55.0	806	51.2	806	89.5	(87.1–91.4)	50.1	0.789	++
Injectable Oxytocin / Ergometrine given during delivery	391	0.8	405	1.0	349	100	–	100	–	
Newborn wrapped before placenta delivered	807	40.6	745	49.1	744	52.2	(48.6–55.7)	50.2	0.040	
Newborn washed before placenta delivered	808	0.1	734	3.5	734	96.3	(94.7–97.5)	96.3	0	
Newborn wiped with cloth before placenta delivered	808	29.8	707	48.2	707	58.0	(54.3–61.6)	50.7	0.148	
Newborn placed on mother’s chest or arms before placenta delivered	807	2.4	780	5.9	779	92.9	(90.9–94.5)	91.9	0.124	
Cord cut after placenta delivered	806	99.4	762	96.5	762	96.1	(94.4–97.2)	95.8	0.052	
Cord cut with new blade	806	98.1	795	99.9	795	98.9	(97.8–99.4)	98.6	0.180	
Anything applied to cord immediately after delivery	807	95.4	788	97.3	788	94.9	(93.2–96.3)	93.1	0.261	
CHX applied to cord stump immediately after delivery	807	94.2	787	96.6	787	94.2	(92.3–95.6)	91.5	0.314	
Breastfeeding initiated in first hour	807	28.1	800	21.3	799	68.1	(64.8–71.2)	62.4	0.151	

**Fig. 2:**
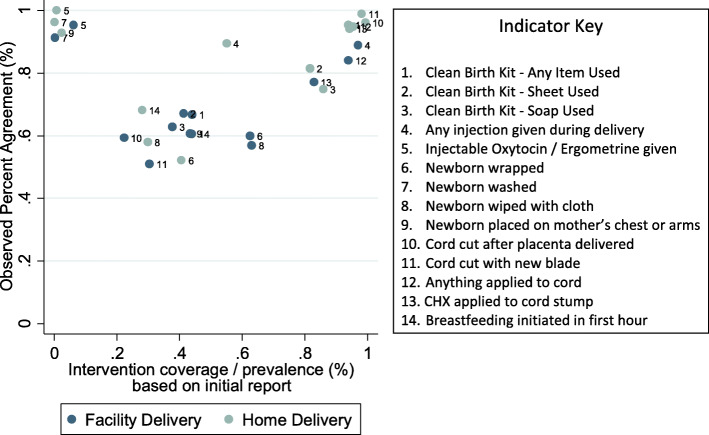
Association between observed percent agreement and intervention coverage / prevalence

**Fig. 3:**
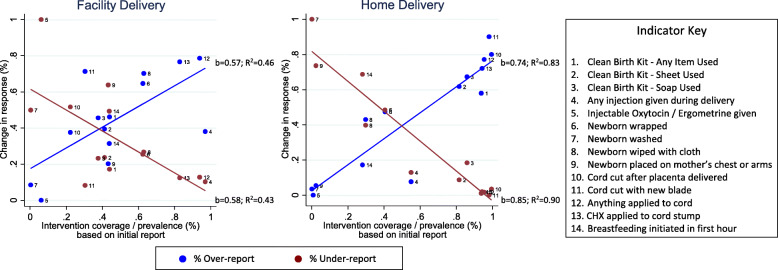
Association between coverage and proportion of women changing response by site of delivery. Significant associations are shown with linear trend and fit metrics

We assessed associations at the indicator level between underlying intervention coverage or prevalence, based on the initial maternal report (after excluding DKs), and measures of maternal recall reliability. There was a U-shaped association between intervention prevalence and observed percent agreement (Fig. 2). The proportion of women who changed their initial report of each indicator and coverage of each intervention or prevalence of each practice in the population is presented in Supplementary Table 4. We observed an association between underlying intervention coverage and the proportion of women over or under-reporting the intervention at the follow-up interview (Fig. 3). Among both home and facility births, we observed a higher proportion of women changed their initial report of no intervention to received intervention (over-reporting) for interventions that were more common in the population. Conversely, we observed a higher proportion of women changed their initial report of received intervention to did NOT receive the intervention (under-reporting) among interventions that were less common in the population. These associations were stronger among home deliveries than facility deliveries. Underlying intervention prevalence accounted for 83% of the variation in over-reporting and 85% of under-reporting among home births, but only 46 and 43% of over- and under-reporting respectively among facility births. There was no association between underlying intervention prevalence and indicator kappa statistics (data not shown).

**Fig. 4:**
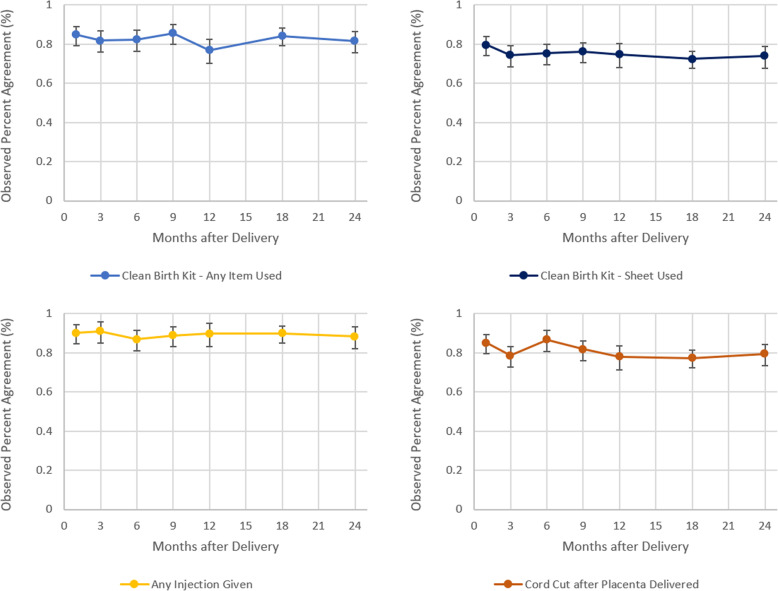
Variation in percent agreement of maternal report by reference period

We observed no significant differences in percent agreement by binned recall time for any of the four best performing indicators (Fig. 4). Similarly, in the adjusted logistic regression model, we observed a negligible but statistically significant reduction in agreement of report by increasing recall period for most indicators, controlling for child sex, place of delivery, birth complications, parity, maternal age, education, and ethnicity (Tables 5). Mothers had statistically lower odds of reliably reporting 10 of 14 interventions if they delivered in a facility and statistically greater odds of reliably reporting if the baby was wrapped before the placenta delivery if they delivered at a facility. After disaggregating by location of delivery, there was little statistical difference in the reliability of report by recall length among home deliveries (Table 6). There were no clear cross-cutting associations between respondent and birth characteristics and over- or under-reporting overall (see Supplementary Tables 5 & 7) or by site of delivery (see Supplementary Tables 6 & 8).

**Table 5:** Characteristics associated with the observed agreement of maternal report

Indicator	n	Recall period (in weeks)		Site of delivery (home [ref] vs facility)		Delivery complications (no complication [ref] vs complications)		Child sex (male [ref] vs female)		Parity		Maternal age (in years)		Maternal education (no education [ref] vs any education)		Maternal ethnicity (Madhesi [ref] vs Pahadi)	
		AOR	95% CI	AOR	95% CI	AOR	95% CI	AOR	95% CI	AOR	95% CI	AOR	95% CI	AOR	95% CI	AOR	95% CI
Clean Birth Kit - Any Item Used	1452	0.998	(0.994–1.002)	0.10	(0.07–0.15)	0.98	(0.69–1.40)	1.16	(0.86–1.57)	0.99	(0.86–1.14)	1.03	(0.98–1.07)	0.99	(0.72–1.37)	0.59	(0.31–1.13)
Clean Birth Kit - Sheet Used	1452	0.997	(0.994–1.001)	0.45	(0.34–0.58)	1.00	(0.72–1.37)	0.94	(0.74–1.20)	0.96	(0.86–1.08)	1.00	(0.97–1.04)	1.04	(0.79–1.38)	0.89	(0.47–1.65)
Clean Birth Kit - Soap Used	1452	0.998	(0.995–1.001)	0.55	(0.43–0.71)	1.13	(0.83–1.54)	0.92	(0.74–1.16)	0.97	(0.87–1.07)	1.02	(0.98–1.05)	1.04	(0.80–1.35)	0.88	(0.49–1.60)
Any injection given	1496	0.999	(0.995–1.004)	0.87	(0.61–1.24)	1.01	(0.65–1.57)	0.80	(0.57–1.11)	1.03	(0.89–1.19)	0.97	(0.93–1.02)	1.24	(0.83–1.85)	1.50	(0.52–4.31)
Injectable Oxytocin / Ergo given*	371																
Newborn wrapped	1331	0.997	(0.994–1.000)	1.38	(1.09–1.76)	0.81	(0.60–1.10)	1.17	(0.94–1.46)	0.97	(0.88–1.08)	1.00	(0.97–1.04)	1.15	(0.89–1.49)	0.82	(0.45–1.49)
Newborn washed	1222	0.995	(0.988–1.001)	0.34	(0.20–0.58)	0.97	(0.53–1.78)	1.81	(1.10–3.00)	0.97	(0.78–1.20)	0.98	(0.91–1.05)	1.20	(0.68–2.11)	–	
Newborn wiped	1245	0.996	(0.993–0.999)	0.97	(0.76–1.24)	0.87	(0.63–1.19)	1.29	(1.02–1.62)	0.96	(0.87–1.07)	1.01	(0.97–1.04)	0.95	(0.72–1.24)	0.98	(0.52–1.83)
Newborn placed on mother’s chest or arms	1435	0.995	(0.992–0.999)	0.12	(0.08–0.16)	0.90	(0.64–1.26)	1.14	(0.86–1.50)	0.90	(0.79–1.02)	1.03	(0.99–1.08)	0.85	(0.63–1.16)	0.78	(0.41–1.48)
Cord cut after placenta delivered	1297	0.995	(0.990–0.999)	0.05	(0.04–0.08)	1.06	(0.72–1.56)	1.02	(0.75–1.41)	1.00	(0.86–1.16)	1.01	(0.96–1.06)	1.23	(0.86–1.76)	2.38	(0.99–5.71)
Cord cut with new blade	1241	0.997	(0.991–1.002)	0.01	(0.01–0.03)	1.03	(0.66–1.59)	0.94	(0.65–1.36)	1.04	(0.87–1.24)	1.00	(0.94–1.06)	0.84	(0.56–1.24)	0.38	(0.16–0.90)
Anything applied to cord	1347	0.994	(0.988–0.999)	0.28	(0.19–0.43)	0.60	(0.39–0.93)	1.15	(0.79–1.68)	0.92	(0.78–1.09)	1.02	(0.97–1.08)	1.24	(0.80–1.92)	0.75	(0.29–1.89)
CHX applied to cord stump	1339	0.99	(0.985–0.995)	0.20	(0.13–0.29)	1.28	(0.82–2.01)	1.05	(0.75–1.48)	1.00	(0.86–1.17)	0.98	(0.94–1.03)	0.82	(0.56–1.20)	0.94	(0.42–2.09)
Breastfeeding in first hour	1487	0.998	(0.995–1.001)	0.71	(0.56–0.90)	1.08	(0.81–1.44)	0.86	(0.69–1.07)	1.05	(0.95–1.16)	0.99	(0.96–1.02)	1.02	(0.79–1.31)	1.47	(0.81–2.66)

* Indicator could not be analyzed due to insufficient variablity in outcome

**Table 6:** Characteristics associated with the observed agreement of maternal report, by site of delivery

	n	Recall period (in weeks)		Delivery complications (none [ref] vs complications)		Child sex (male [ref] vs female)		Parity		Maternal age (in years)		Maternal education (no education [ref] vs any education)		Maternal ethnicity (Madhesi [ref] vs Pahadi)	
		AOR	95% CI	AOR	95% CI	AOR	95% CI	AOR	95% CI	AOR	95% CI	AOR	95% CI	AOR	95% CI
**Facility Delivery**															
Clean Birth Kit - Any Item Used	649	0.997	(0.993–1.002)	1.00	(0.68–1.45)	1.22	(0.87–1.71)	1.00	(0.85–1.17)	1.01	(0.96–1.06)	0.88	(0.62–1.26)	0.54	(0.27–1.08)
Clean Birth Kit - Sheet Used	649	0.995	(0.990–1.000)	1.01	(0.69–1.46)	1.16	(0.83–1.62)	1.00	(0.85–1.17)	1	(0.95–1.05)	0.95	(0.67–1.36)	0.73	(0.36–1.48)
Clean Birth Kit - Soap Used	649	0.998	(0.993–1.002)	1.16	(0.80–1.68)	1.07	(0.77–1.48)	0.91	(0.78–1.07)	1.05	(0.99–1.10)	0.89	(0.63–1.26)	0.76	(0.38–1.52)
Any injection given	692	1.004	(0.997–1.011)	1.44	(0.82–2.55)	0.86	(0.53–1.38)	1.10	(0.89–1.35)	0.94	(0.88–1.00)	1.11	(0.66–1.87)	2.56	(0.59–11.15)
Injectable Oxytocin / Ergo given*	22														
Newborn wrapped	589	0.994	(0.989–0.999)	0.85	(0.58–1.24)	0.97	(0.70–1.36)	1.07	(0.91–1.27)	0.98	(0.93–1.03)	1.11	(0.77–1.60)	0.81	(0.39–1.67)
Newborn washed	505	0.997	(0.989–1.006)	1.51	(0.71–3.21)	2.41	(1.24–4.69)	0.90	(0.68–1.18)	0.98	(0.89–1.08)	1.90	(0.93–3.92)	–	
Newborn wiped	540	0.994	(0.989–0.999)	0.77	(0.52–1.13)	1.16	(0.82–1.64)	1.01	(0.85–1.20)	1.00	(0.95–1.06)	1.03	(0.70–1.49)	1.15	(0.53–2.51)
Newborn placed on mother’s chest or arms	658	0.994	(0.989–0.998)	0.94	(0.66–1.35)	1.11	(0.80–1.53)	0.87	(0.74–1.01)	1.02	(0.97–1.07)	0.72	(0.51–1.02)	0.78	(0.39–1.57)
Cord cut after placenta delivered	537	0.996	(0.991–1.001)	1.02	(0.68–1.54)	1.01	(0.71–1.44)	0.99	(0.84–1.17)	1.00	(0.95–1.06)	1.13	(0.77–1.65)	2.85	(1.12–7.26)
Cord cut with new blade	448	0.996	(0.991–1.002)	1.04	(0.67–1.63)	0.90	(0.61–1.31)	1.06	(0.88–1.28)	0.99	(0.93–1.06)	0.80	(0.53–1.20)	0.36	(0.14–0.89)
Anything applied to cord	560	0.996	(0.989–1.003)	0.60	(0.36–0.98)	1.26	(0.79–2.00)	0.98	(0.79–1.22)	1.01	(0.94–1.08)	1.41	(0.84–2.35)	0.60	(0.23–1.61)
CHX applied to cord stump	553	0.989	(0.983–0.995)	1.40	(0.85–2.30)	1.39	(0.92–2.11)	1.09	(0.89–1.32)	0.97	(0.91–1.03)	0.79	(0.51–1.23)	1.02	(0.42–2.47)
Breastfeeding in first hour	690	0.998	(0.993–1.002)	1.01	(0.72–1.42)	0.90	(0.66–1.23)	1.07	(0.93–1.24)	0.98	(0.94–1.03)	1.03	(0.74–1.44)	1.43	(0.71–2.90)
**Home Delivery**															
Clean Birth Kit - Any Item Used	786	1.001	(0.992–1.011)	0.85	(0.29–2.50)	0.96	(0.49–1.87)	0.93	(0.67–1.29)	1.10	(0.98–1.24)	2.10	(0.79–5.61)	–	
Clean Birth Kit - Sheet Used	803	1	(0.995–1.005)	1.03	(0.54–1.95)	0.73	(0.51–1.04)	0.93	(0.79–1.08)	1.01	(0.96–1.07)	1.25	(0.77–2.02)	3.14	(0.41–24.25)
Clean Birth Kit - Soap Used	803	0.998	(0.994–1.003)	1.02	(0.58–1.81)	0.79	(0.58–1.09)	1.01	(0.88–1.17)	0.99	(0.95–1.04)	1.29	(0.84–1.97)	1.42	(0.40–5.12)
Any injection given	804	0.996	(0.990–1.002)	0.56	(0.28–1.09)	0.77	(0.49–1.21)	0.97	(0.80–1.19)	1.00	(0.94–1.07)	1.57	(0.82–3.00)	0.65	(0.14–3.02)
Injectable Oxytocin / Ergo given*	349														
Newborn wrapped	742	0.999	(0.994–1.003)	0.77	(0.45–1.30)	1.34	(1.00–1.79)	0.92	(0.80–1.05)	1.02	(0.97–1.06)	1.23	(0.84–1.79)	0.91	(0.32–2.59)
Newborn washed	717	0.991	(0.980–1.002)	0.32	(0.12–0.84)	1.22	(0.55–2.70)	1.21	(0.82–1.77)	0.95	(0.84–1.06)	0.47	(0.20–1.15)	–	
Newborn wiped	705	0.997	(0.993–1.001)	1.17	(0.67–2.03)	1.40	(1.03–1.89)	0.93	(0.81–1.07)	1.01	(0.97–1.06)	0.88	(0.60–1.30)	0.64	(0.21–1.98)
Newborn placed on mother’s chest or arms	777	1.001	(0.993–1.009)	0.59	(0.25–1.39)	1.26	(0.72–2.22)	0.95	(0.73–1.24)	1.09	(0.99–1.19)	1.70	(0.79–3.66)	0.78	(0.10–6.31)
Cord cut after placenta delivered	760	0.991	(0.981–1.001)	1.34	(0.31–5.85)	1.16	(0.55–2.45)	1.02	(0.71–1.45)	1.06	(0.94–1.19)	2.23	(0.73–6.78)	0.42	(0.05–3.56)
Cord cut with new blade	776	0.998	(0.980–1.017)	0.73	(0.09–6.09)	1.72	(0.42–6.98)	0.84	(0.47–1.50)	1.09	(0.88–1.36)	2.33	(0.28–19.59)	–	
Anything applied to cord	770	0.99	(0.981–0.998)	0.69	(0.26–1.85)	0.97	(0.51–1.85)	0.83	(0.63–1.09)	1.04	(0.95–1.15)	0.96	(0.42–2.21)	–	
CHX applied to cord stump	786	0.992	(0.984–1.000)	1.06	(0.36–3.09)	0.58	(0.32–1.07)	0.86	(0.67–1.11)	1.02	(0.94–1.12)	1.02	(0.46–2.27)	0.89	(0.11–7.32)
Breastfeeding in first hour	797	0.999	(0.995–1.003)	1.31	(0.75–2.29)	0.82	(0.61–1.11)	1.03	(0.90–1.18)	1.00	(0.96–1.05)	1.01	(0.69–1.48)	1.62	(0.51–5.13)

* Indicator could not be analyzed due to insufficient variability in outcome

## Discussion

This study found poor reliability of maternal report of immediate newborn care indicators as collected through a household survey. A high percent agreement between initial and follow-up reports was observed for most interventions. However, the kappa values for most interventions were low, suggesting observed agreement was mostly due to chance from very high or very low intervention coverage. This was further evidenced by the U-shaped association between intervention prevalence and observed agreement. Only receipt of an injection during delivery could be recalled with high reliability, but not information on the type of injection. For most indicators, women who delivered at home had greater odds of reliably reporting on the intervention compared to women who delivered at a facility. We also observed a high proportion of women who delivered in health facilities failing to recall interventions during the initial interview (< 72 h) after delivery. A negligible, although sometimes statistically significant, decline in recall reliability with increasing time since birth was observed for most indicators.

This study reinforces previous research that suggests mothers are unable to effectively recall interventions or practices which occur during the peripartum period. Studies by Blanc, McCarthy, and Stanton have demonstrated poor recall accuracy within the peripartum period among women in multiple low- and middle-income settings [2–6]. Multiple factors could contribute to poor recall. Our study suggests that mothers may never have known about some interventions they received – as evidenced by the high proportion of mothers that reported they didn’t know the type of injection they received among both facility and home births at the initial assessment. This agrees with findings from Mexico and Kenya, which showed poor recall of peripartum interventions among women at initial discharge from their labor and delivery facility [2, 3]. Similar to previous studies, we found recall close to the time of care was generally poor; however, there was little evidence of reliability-altering recall degradation with increasing time since delivery up to a two-year recall period [5, 6]. Currently, the DHS asks women to report on delivery and newborn care for their most recent birth in the previous 3 years, with surveys prior to Phase 8 asking about births in the previous 5 years. However, this study and others only assessed recall for up to 2 years postpartum.

Previous studies have primarily assessed recall among women who delivered in health facilities. In general, this study found the proportion of women who were unable to report whether they received an intervention within 72 h of birth was higher among facility deliveries relative to home deliveries. A possible explanation is that women delivering in a facility may not have been informed about the details of interventions received, such as the substance applied to the child’s umbilical cord or whether the instrument used to cut the cord was new or had been sterilized. We also observed significantly weaker recall reliability for most indicators among women who delivered at a facility compared to women who delivered at home. This suggests receipt of these interventions were less salient events for women delivering in facilities potentially because they were not informed or counseled on various interventions, events may have been obscured by an unfamiliar or chaotic environment, or they paid less attention to events because they trusted the skilled attendants providing care. Alternatively, mothers may have had a better rapport with home birth attendants, often family or other community members, who may have more effectively communicated events occurring throughout the delivery process. No other characteristics had a consistent effect on mothers’ recall reliability.

We observed an association between underlying intervention coverage and maternal report. Interventions that were more common in the population were more likely to be over-reported during the follow-up interview. Likewise, interventions that were uncommon in the population were more likely to be under-reported. This association was stronger among home births than facility births. A forthcoming assessment of maternal report of antenatal and postnatal care interventions in Kenya, Cambodia, and Bangladesh found a similar association between intervention prevalence and under- and over-reporting of intervention receipt among women receiving facility-based care [8]. This association could potentially reflect a social desirability bias, such as wanting to report a practice the mother thought the interviewer would perceive favorably. Alternatively, if the mother could not clearly recall the intervention, she may assume that an intervention did or did not occur if it was or was not a standard practice in the setting.

This study had a number of limitations. This analysis is limited to assessing the test-retest reliability of maternal report and changes in response over time. We were unable to observe interventions and practices during delivery, so we are unable to assess the validity of maternal report. Our assessment did effectively assess changes in recall over time and demonstrated inconsistencies in women’s reports of interventions received. Another limitation is that women were classified as having delivered at home or in a facility; however, 2.3% of women delivered on the way to a health facility. These women were classified as delivering at home because of the lack of services they would have received in-transit. Less than half (*n* = 11) of those women reported continuing on to a health facility, so we would expect that categorizing them as home births would have minimal effect on the study findings. Additionally, this study was conducted in a study population that has received a number of interventions related to clean delivery and newborn care over a number of years. This population may have been uniquely primed to recall interventions or may have felt additional pressure to report use of specific interventions. Use of clean delivery kits and clean cord practices was high in this population among both home and facility births due to the trial protocol of providing clean delivery kits and the existence of a successful national chlorhexidine cord care program in Nepal. The reliability of reporting may be different within a population with lower coverage and perhaps less awareness of these interventions. Additional work is needed to assess coverage of interventions among home births in populations without high access to clean birth kits and programs for safer home delivery practices.

## Conclusions

This study reinforces previous findings that mothers are unable to effectively report on many interventions or practices within the peripartum period. Household surveys which rely on maternal report therefore may not be an appropriate method for collecting data on coverage of many interventions during the peripartum period. This is particularly true among facility births, where many interventions may occur without the mother’s full knowledge. New methods are needed for generating more robust estimates of peripartum intervention coverage. Data suggest that mothers are able to accurately report on the location on delivery. Linking valid data on where mothers deliver with robust data on the content and quality of delivery care at these facilities may be used to generate estimates of intervention coverage.

## Supplementary Information

**Additional file 1: Supplementary Questionnaire 1.** Questions used in initial survey. **Supplementary Questionnaire 2.** Questions used in follow-up survey. **Supplementary Figure 1.** Flowchart of participant selection; **Supplementary Table 1.** Characteristics of sample, stratified by site of delivery. **Supplementary Table 2.** Reliability of maternal report of immediate newborn care indicators treating “don’t know” as response category. **Supplementary Table 3.** Reliability of maternal report of immediate newborn care indicators treating “don’t know” as response category, by site of delivery. **Supplementary Table 4.** Coverage of intervention and measures of maternal report, by site of delivery. **Supplementary Table 5.** Characteristics associated with maternal over-reporting. **Supplementary Table 6.** Characteristics associated with maternal over-reporting, by site of delivery. **Supplementary Table 7.** Characteristics associated with maternal under-reporting. **Supplementary Table 8.** Characteristics associated with maternal under-reporting, by site of delivery

## Data Availability

The datasets used and/or analysed during the current study are available from the corresponding author on reasonable request.

## Abbreviations

CHX: Chlorhexidine
DHS: Demographic and Health Survey
DK: Don’t know
p_e_: Expected percent agreement
κ: Kappa
MICS: Multiple Indicator Cluster Survey
NNIPS: Nepal Nutrition Intervention Project – Sarlahi
p_o_: Observed percent agreement
