# Supplementary material for: Reliability of maternal recall of delivery and immediate newborn care indicators in Sarlahi, Nepal
Source: BMC Pregnancy Childbirth. 2021 Jan 25;21:82. doi: 10.1186/s12884-021-03547-5 (PMC7831166; doi:10.1186/s12884-021-03547-5)
Supplement: Supplementary file 1 — Additional file 1: Supplementary Questionnaire 1. Questions used in initial survey. Supplementary Questionnaire 2. Questions used in follow-up survey. Supplementary Figure 1. Flowchart of participant selection; Supplementary Table 1. Characteristics of sample, stratified by site of delivery. Supplementary Table 2. Reliability of maternal report of immediate newborn care indicators treating “don’t know” as response category. Supplementary Table 3. Reliability of maternal report of immediate newborn care indicators treating “don’t know” as response category, by site of delivery. Supplementary Table 4. Coverage of intervention and measures of maternal report, by site of delivery. Supplementary Table 5. Characteristics associated with maternal over-reporting. Supplementary Table 6. Characteristics associated with maternal over-reporting, by site of delivery. Supplementary Table 7. Characteristics associated with maternal under-reporting. Supplementary Table 8. Characteristics associated with maternal under-reporting, by site of delivery [file 12884_2021_3547_MOESM1_ESM.pdf]

Supplementary material for: Reliability of maternal recall of delivery and immediate newborn care indicators in Sarlahi, Nepal

Supplemental File 1. Questions used in initial survey

Where did the delivery take place?
1=At home
2=Maiti
3=At health post/clinic
4=In hospital
5=On the way to facility
6=Outdoors
9=Don't Know

Were any injections/saline given to the mother before, during or after labor?
0=No
1=Yes
9=Don't know

What type of injection/saline was given?
1=Oxytocin
2=Ergometrine
3=Vitamins
4=Antibiotic
5=Saline IV drip
6=Other, specify
9=Don't Know

Was any part of the clean birthing kit used for the delivery?
0=No
1=Yes
9=Don't know

Was the soap used to wash hands before delivery by the person who delivered the baby?
0=No
1=Yes
9=Don't know

Was the string used to tie the cord?
0=No
1=Yes
9=Don't know

Was the blade used to cut the cord?
0=No
1=Yes
9=Don't know

Was the plastic disc used when cutting the cord?
- 0=No
- 1=Yes
- 9=Don't know

Was the plastic sheet kept under the woman during delivery?
- 0=No
- 1=Yes
- 9=Don't know

During the time before the placenta came out, how was the baby cared for:

- Was the child wiped with a cloth before the placenta came out?
  - 0=No
  - 1=Yes
  - 9=Don't know

- Was the child washed with water before the placenta came out?
  - 0=No
  - 1=Yes
  - 9=Don't know

- Was the child wrapped in a blanket before the placenta came out?
  - 0=No
  - 1=Yes
  - 9=Don't know

- Was the child massaged with oil before the placenta came out?
  - 0=No
  - 1=Yes
  - 9=Don't know

Where was the baby placed before the placenta came out?
- 1 = Floor
- 2 = Bed
- 3 = Mother's Arms/Chest
- 4 = Other caretaker's arms
- 9 = Don't Know

Was the baby's cord cut before or after the placenta came out?
- 1=Before
- 2=After
- 9=Don't Know

What was used to cut the cord?

1= New blade
2= Other blade
3= Household knife
4= Sickle
5= Other, specify
9= Don't Know

Was anything applied to the cord immediately after cutting?

0=No
1=Yes
9=Don't know

What was applied to the cord?

1=Mustard oil
2=Sunflower oil
3=Ash
4=Mud
5=CHX from Kawach Tube
6=Other Antiseptic (eg. Betadine, Dettol)
7=Herbs/spices
8= Other, specify
9=Don't Know

Has the baby been breastfed since birth?

0=No
1=Yes
9=Don't know

How many hours passed after birth before breastfeeding was initiated?

00=< 1 hour
01-98 = hours
99= Don't Know

Supplemental File 2. Questions used in follow-up survey

Did you ever breastfeed (NAME)?
- 0=No
- 1=Yes
- 9=Don't know

How long after birth did you first put (NAME) to the breast?
- IF LESS THAN 1 HOUR, RECORD '00' HOURS AND '0=IMMEDIATELY'; RECORD THE HOURS OR DAYS THE WOMAN REPORTS.
- 99= Don't Know

Were any injections/saline given to you before, during or after labor?
- 0=No
- 1=Yes
- 9=Don't know

What type of injection/saline was given?
- 1=Oxytocin
- 2=Ergometrine
- 3=Vitamins
- 4=Antibiotic
- 5=Saline IV drip
- 6=Other, specify
- 9 = Don't know

Was any part of the clean birthing kit used for the delivery?
- 0=No
- 1=Yes
- 9=Don't know

Was the soap used to wash hands before delivery by the person who delivered your child?
- 0=No
- 1=Yes
- 9=Don't know

Was the string used to tie the cord?
- 0=No
- 1=Yes
- 9=Don't know

Was the blade used to cut the cord?
- 0=No
- 1=Yes
- 9=Don't know

Was the plastic disc used when cutting the cord?

- 0=No
- 1=Yes
- 9=Don't know

Was the plastic sheet kept under the woman during delivery?

- 0=No
- 1=Yes
- 9=Don't know

Were the gloves used when delivering the baby?

- 0=No
- 1=Yes
- 9=Don't know

I would now like to ask you about how the child was cared for during the time before the placenta came out:

- Was the child wiped with a cloth before the placenta came out?
  - 0=No
  - 1=Yes
  - 9=Don't know

- Was the child washed with water before the placenta came out?
  - 0=No
  - 1=Yes
  - 9=Don't know

- Was the child wrapped in a blanket before the placenta came out?
  - 0=No
  - 1=Yes
  - 9=Don't know

- Was the child massaged with oil before the placenta came out?
  - 0=No
  - 1=Yes
  - 9=Don't know

Where was the child placed before the placenta came out?

- 1 = Floor
- 2 = Bed
- 3 = Mother's Arms/Chest
- 4 = Other caretaker's arms
- 9 = Don't Know

Was the child's cord cut before or after the placenta came out?

- 1=Before
- 2=After
- 9=Don't Know

What was used to cut the cord?

1= New blade
2= Other blade
3= Household knife
4= Sickle
5= Other, specify
9= Don't Know

Was anything applied to the cord immediately after cutting?

0=No
1=Yes
9=Don't know

What was applied to the cord? Anything else? RECORD ALL THAT WAS APPLIED.

1=Mustard oil
2=Sunflower oil
3=Ash
4=Mud
5=CHX from Kawach Tube
6=Other Antiseptic (eg. Betadine, Dettol)
7=Herbs/spices
8=Other, specify
9=Don't Know

Supplemental Figure 1. Flowchart of participant selection

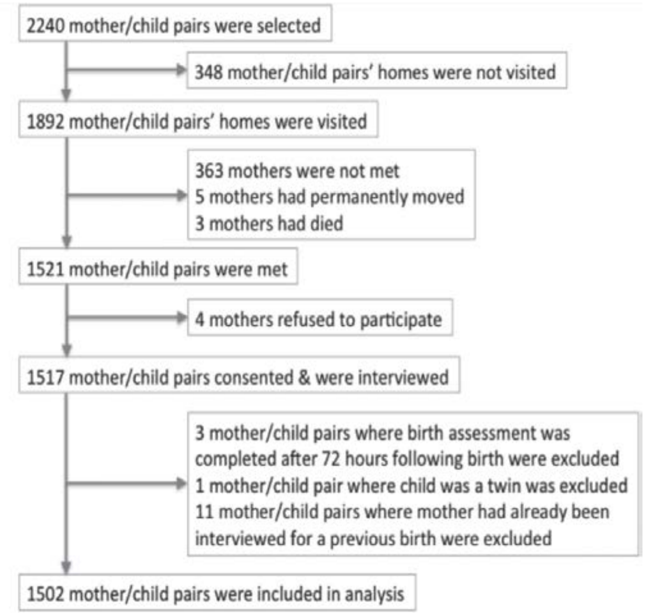

Supplemental Table 1. Characteristics of sample, stratified by site of delivery

		Facility			Home		
		n	Mean / %	95% CI	n	Mean / %	95% CI
**Recall period (weeks)**		693	46.1	(43.5, 48.6)	808	47.8	(45.4, 50.2)
**Child sex**	Male	387	55.8	(52.1, 59.5)	447	55.3	(51.9, 58.7)
	Female	306	44.2	(40.5, 47.9)	361	44.7	(41.3, 48.1)
**Birth complications**	None	492	71.1	(67.6, 74.4)	738	91.3	(89.2, 93.1)
	Reported	200	28.9	(25.6, 32.4)	70	8.7	(6.9, 10.8)
**Parity**	Primiparous	288	41.6	(37.9, 45.3)	140	17.3	(14.9, 20.1)
	Second	172	24.8	(21.7, 28.2)	210	26	(23.1, 29.1)
	Third	117	16.9	(14.3, 19.9)	194	24	(21.2, 27.1)
	Fourth +	116	16.7	(14.1, 19.7)	264	32.7	(29.5, 36)
**Maternal Age (yrs) at delivery**	<20	197	28.4	(25.2, 31.9)	151	18.7	(16.1, 21.5)
	20-29	429	61.9	(58.2, 65.5)	548	67.8	(64.5, 71)
	30-39	61	8.8	(6.9, 11.2)	101	12.5	(10.4, 15)
	40+	6	0.9	(0.4, 1.9)	8	1	(0.5, 2)
**Maternal education**	None	394	56.9	(53.1, 60.5)	631	78.2	(75.2, 80.9)
	Any	299	43.1	(39.5, 46.9)	176	21.8	(19.1, 24.8)
**Ethnicity**	Madhesi	653	94.2	(92.2, 95.7)	790	97.9	(96.6, 98.7)
	Pahadi	40	5.8	(4.3, 7.8)	17	2.1	(1.3, 3.4)
**HH electricity**	No electricity	107	15.4	(12.9, 18.3)	186	23	(20.3, 26.1)
	Had electricity	586	84.6	(81.7, 87.1)	621	77	(73.9, 79.7)
**HH latrine status**	No latrine	448	64.6	(61, 68.1)	619	76.7	(73.7, 79.5)
	Had latrine	245	35.4	(31.9, 39)	188	23.3	(20.5, 26.3)
**Land ownership**	Did not own land	15	2.2	(1.3, 3.6)	24	3	(2, 4.4)
	Owns land	678	97.8	(96.4, 98.7)	783	97	(95.6, 98)

Supplemental Table 2. Reliability of maternal report of immediate newborn care indicators treating “don’t know” as response category

	Proportion report receiving intervention						Observed Agreement			Expected Agreement	Kappa	K + >0.4 ++ >0.6
	Initial			Follow-up								
	n	% Yes	% Miss	n	% Yes	% Miss	n	%	95% CI	%		
Clean Birth Kit - Any Item Used	1500	70.3	1.2	1502	79.4	1.9	1500	80.3	(78.2-82.2)	61.2	0.492	+
Clean Birth Kit - Sheet Used	1500	62.3	1.4	1502	68.0	4.3	1500	71.7	(69.4-74.0)	52.5	0.405	+
Clean Birth Kit - Soap Used	1500	60.7	3.7	1502	65.0	5.0	1500	63.8	(61.3-66.2)	50.4	0.271	
Any injection given during delivery	1502	74.4	0.0	1502	68.2	0.1	1502	89.1	(87.4-90.6)	58.9	0.735	++
Injectable Oxytocin / Ergometrine given during delivery	1502	0.4	70.8	1502	0.4	65.2	1502	85.3	(83.4-87.0)	56.1	0.665	++
Newborn wrapped before placenta delivered	1502	50.1	1.1	1502	52.9	10.3	1502	49.8	(47.3-52.3)	44.6	0.095	
Newborn washed before placenta delivered	1502	0.2	1.0	1502	4.9	14.4	1502	80.4	(78.3-82.3)	79.9	0.024	
Newborn wiped with cloth before placenta delivered	1502	44.2	1.5	1502	49.5	15.8	1502	48.3	(45.8-50.9)	41	0.125	
Newborn placed on mother’s chest or arms before placenta delivered	1502	20.9	0.9	1502	15.1	3.4	1502	75	(72.7-77.1)	66.9	0.243	
Cord cut after placenta delivered	1502	63.1	2.3	1502	63.7	11.9	1502	70.8	(68.5-73.1)	54.2	0.334	
Cord cut with new blade	1502	65.2	5.0	1502	78.8	14.1	1502	69.5	(67.1-71.8)	69	0.311	
Anything applied to cord immediately after delivery	1502	92.3	2.5	1502	85.0	8.5	1502	82.1	(80.1-83.9)	79	0.147	
CHX applied to cord stump immediately after delivery	1502	86.8	0.1	1502	84.5	8.9	1502	78.4	(76.2-80.4)	74.2	0.162	
Breastfeeding initiated in first hour	1502	35.4	0.1	1502	29.8	0.6	1502	64.2	(61.7-66.6)	55.4	0.196	

Supplemental Table 3. Reliability of maternal report of immediate newborn care indicators treating "don't know" as response category, by site of delivery

Facility Delivery	Proportion report receiving intervention						Observed Agreement			Expected Agreement	Kappa	K + >0.4 ++ >0.6
	Initial			Follow-up								
	n	% Yes	% Miss	n	% Yes	% Miss	n	%	95% CI	%		
Clean Birth Kit - Any Item Used	691	42.8	2.6	693	59.9	3.8	691	63.1	(59.4-66.6)	45.6	0.321	
Clean Birth Kit - Sheet Used	691	39.9	2.9	693	48.8	7.5	691	61.5	(57.8-65.1)	44.7	0.303	
Clean Birth Kit - Soap Used	691	32.1	7.2	693	50.6	8.1	691	53.7	(50.0-57.4)	41.9	0.203	
Any injection given during delivery	693	97.0	0.0	693	88.2	0.0	693	88.9	(86.3-91.0)	85.9	0.215	
Injectable Oxytocin / Ergometrine given during delivery	693	0.4	93.1	693	0.3	83.0	693	82.3	(79.2-84.9)	78.3	0.182	
Newborn wrapped before placenta delivered	693	61.0	2.3	693	61.6	13.3	693	51.8	(48.1-55.5)	47.1	0.089	
Newborn washed before placenta delivered	693	0.3	2.2	693	6.9	20.5	693	72	(68.5-75.2)	71.3	0.026	
Newborn wiped with cloth before placenta delivered	693	60.9	3.3	693	58.0	19.6	693	45.5	(41.8-49.2)	44	0.026	
Newborn placed on mother's chest or arms before placenta delivered	693	42.6	1.7	693	26.1	3.3	693	57.9	(54.1-61.5)	50.5	0.149	
Cord cut after placenta delivered	693	21.2	4.8	693	32.0	19.0	693	47.5	(43.8-51.2)	43.9	0.063	
Cord cut with new blade	693	27.3	10.4	693	56.1	28.7	693	36.9	(33.4-40.6)	27.7	0.127	
Anything applied to cord immediately after delivery	693	88.9	5.3	693	73.3	15.4	693	69.7	(66.2-73.0)	66.6	0.092	
CHX applied to cord stump immediately after delivery	693	78.2	0.3	693	72.4	16.3	693	62.5	(58.8-66.0)	59.1	0.082	
Breastfeeding initiated in first hour	693	43.9	0.1	693	40.0	0.1	693	60.5	(56.8-64.0)	51.1	0.192	
Home Delivery												
Clean Birth Kit - Any Item Used	808	93.8	0.0	808	96.0	0.4	808	95	(93.3-96.3)	90.3	0.489	+
Clean Birth Kit - Sheet Used	808	81.4	0.1	808	84.4	1.5	808	80.6	(77.7-83.2)	71.3	0.322	
Clean Birth Kit - Soap Used	808	85.1	0.7	808	77.4	2.4	808	72.5	(69.3-75.5)	68.7	0.121	
Any injection given during delivery	808	55.0	0.0	808	51.1	0.2	808	89.2	(86.9-91.2)	50	0.785	++
Injectable Oxytocin / Ergometrine given during delivery	808	0.4	51.6	808	0.5	49.9	808	87.9	(85.4-89.9)	49.6	0.759	++
Newborn wrapped before placenta delivered	808	40.6	0.1	808	45.3	7.8	808	48	(44.6-51.5)	46.2	0.034	
Newborn washed before placenta delivered	808	0.1	0.0	808	3.2	9.2	808	87.5	(85.0-89.6)	87.5	-0.002	
Newborn wiped with cloth before placenta delivered	808	29.8	0.0	808	42.2	12.5	808	50.7	(47.3-54.2)	44.4	0.114	
Newborn placed on mother's chest or arms before placenta delivered	808	2.4	0.1	808	5.7	3.5	808	89.6	(87.3-91.5)	88.7	0.077	
Cord cut after placenta delivered	808	99.1	0.2	808	91.0	5.7	808	90.8	(88.6-92.6)	90.2	0.064	
Cord cut with new blade	808	97.9	0.2	808	98.3	1.6	808	97.5	(96.2-98.4)	96.2	0.348	
Anything applied to cord immediately after delivery	808	95.3	0.1	808	94.9	2.5	808	92.7	(90.7-94.3)	90.6	0.225	
CHX applied to cord stump immediately after delivery	808	94.1	0.0	808	94.8	2.6	808	92	(89.9-93.6)	89.3	0.246	
Breastfeeding initiated in first hour	808	28.1	0.1	808	21.0	1.0	808	67.3	(64.0-70.5)	61.9	0.143	

Supplemental Table 4. Coverage of intervention and measures of maternal report, by site of delivery

Facility Delivery	% Women Initially Reporting Intervention		Over-report (% Changed Response No -> Yes)		Under-report (% Changed Response Yes -> No)	
	%	95% CI	%	95% CI	%	95% CI
Clean Birth Kit - Any Item Used	44	(40.3-47.8)	46.2	(41.1-51.4)	17.2	(13.3-22.0)
Clean Birth Kit - Sheet Used	41.3	(37.6-45.1)	39.4	(34.5-44.4)	23.7	(19.0-29.1)
Clean Birth Kit - Soap Used	37.7	(34.2-41.5)	45.6	(40.8-50.5)	23.3	(18.4-29.0)
Any injection given during delivery	97	(95.4-98.0)	38.1	(20.0-60.3)	10.3	(8.2-12.8)
Injectable Oxytocin / Ergometrine given during delivery	6.2	(1.9-18.3)	0	-	100	-
Newborn wrapped before placenta delivered	62.5	(58.8-66.1)	64.7	(58.1-70.8)	25.5	(21.4-30.2)
Newborn washed before placenta delivered	0.3	(0.1-1.2)	8.5	(6.5-11.2)	50	(1.9-98.1)
Newborn wiped with cloth before placenta delivered	63	(59.3-66.6)	70.3	(63.6-76.2)	26.8	(22.4-31.8)
Newborn placed on mother's chest or arms before placenta delivered	43.3	(39.6-47.1)	20.2	(16.4-24.6)	63.9	(58.2-69.2)
Cord cut after placenta delivered	22.3	(19.3-25.6)	37.6	(33.1-42.4)	51.7	(42.7-60.6)
Cord cut with new blade	30.4	(26.9-34.2)	71.4	(65.9-76.3)	8.2	(4.8-13.6)
Anything applied to cord immediately after delivery	93.9	(91.8-95.5)	78.6	(59.4-90.2)	12.8	(10.2-15.9)
CHX applied to cord stump immediately after delivery	82.9	(79.8-85.6)	75	(65.1-82.8)	12.1	(9.4-15.4)
Breastfeeding initiated in first hour	43.9	(40.3-47.7)	31.5	(27.1-36.3)	49.3	(43.7-55.0)
Home Delivery						
Clean Birth Kit - Any Item Used	93.8	(91.9-95.3)	58	(43.9-70.9)	1.1	(0.5-2.1)
Clean Birth Kit - Sheet Used	81.6	(78.7-84.1)	61.7	(53.7-69.2)	8.6	(6.7-11.1)
Clean Birth Kit - Soap Used	85.9	(83.3-88.1)	67.3	(58.1-75.3)	18.3	(15.6-21.4)
Any injection given during delivery	55	(51.5-58.4)	7.7	(5.4-10.9)	12.9	(10.1-16.4)
Injectable Oxytocin / Ergometrine given during delivery	0.8	(0.2-2.4)	0		-	
Newborn wrapped before placenta delivered	40.6	(37.3-44.1)	47.4	(42.8-52.1)	48.5	(42.9-54.2)
Newborn washed before placenta delivered	0.1	(0.0-0.9)	3.5	(2.4-5.2)	100	-
Newborn wiped with cloth before placenta delivered	29.8	(26.8-33.1)	43	(38.7-47.4)	39.7	(33.4-46.4)
Newborn placed on mother's chest or arms before placenta delivered	2.4	(1.5-3.7)	5.4	(4.0-7.2)	73.7	(49.5-88.9)
Cord cut after placenta delivered	99.4	(98.5-99.7)	80	(25.7-97.9)	3.4	(2.3-5.0)
Cord cut with new blade	98.1	(96.9-98.9)	90	(50.5-98.8)	0	-
Anything applied to cord immediately after delivery	95.4	(93.7-96.7)	77.1	(60.3-88.3)	1.7	(1.0-3.0)
CHX applied to cord stump immediately after delivery	94.2	(92.3-95.6)	72.1	(56.8-83.5)	2	(1.2-3.3)
Breastfeeding initiated in first hour	28.1	(25.1-31.3)	17.3	(14.4-20.6)	68.7	(62.4-74.4)

Supplemental Table 5. Characteristics associated with maternal over-reporting

Indicator	n	Recall period (in weeks)		Site of delivery (home [ref] vs facility)		Delivery complications (no complication [ref] vs complications)		Child sex (male [ref] vs female)		Parity		Maternal age (in years)		Maternal education (no education [ref] vs any education)		Maternal ethnicity (Madhesi [ref] vs Pahadi)	
		AOR	95% CI	AOR	95% CI	AOR	95% CI	AOR	95% CI	AOR	95% CI	AOR	95% CI	AOR	95% CI	AOR	95% CI
Clean Birth Kit - Any Item Used	408	1.004	(0.997-1.010)	0.68	(0.36-1.26)	0.61	(0.39-0.96)	0.95	(0.63-1.42)	0.95	(0.78-1.15)	0.99	(0.93-1.05)	0.78	(0.51-1.19)	1.06	(0.48-2.32)
Clean Birth Kit - Sheet Used	523	1.005	(0.999-1.010)	0.51	(0.34-0.78)	0.65	(0.42-1.00)	1.15	(0.80-1.65)	0.99	(0.84-1.17)	1.01	(0.96-1.07)	0.66	(0.45-0.98)	0.84	(0.38-1.86)
Clean Birth Kit - Soap Used	513	1.002	(0.996-1.008)	0.52	(0.32-0.83)	0.6	(0.39-0.91)	1.17	(0.81-1.68)	1.08	(0.91-1.28)	0.97	(0.92-1.02)	0.81	(0.54-1.20)	0.7	(0.31-1.55)
Any injection given	384	1.006	(0.995-1.017)	7.71	(2.68-22.19)	3.14	(0.93-10.52)	1.17	(0.56-2.42)	0.83	(0.60-1.16)	1.04	(0.94-1.14)	1.17	(0.48-2.89)	0.3	(0.03-3.02)
Injectable Oxytocin / Ergo given	370																
Newborn wrapped	660	1.003	(0.998-1.007)	2.11	(1.45-3.07)	0.92	(0.59-1.46)	0.69	(0.51-0.95)	0.99	(0.85-1.14)	1	(0.95-1.04)	0.94	(0.64-1.38)	1.09	(0.44-2.68)
Newborn washed	1219	1.006	(0.999-1.012)	2.95	(1.74-5.01)	1.06	(0.58-1.96)	0.54	(0.32-0.90)	1.04	(0.84-1.29)	1.02	(0.95-1.10)	0.87	(0.50-1.54)	1	-
Newborn wiped	694	1.004	(1.000-1.009)	3.4	(2.28-5.05)	0.73	(0.45-1.18)	0.59	(0.43-0.82)	0.97	(0.84-1.12)	0.99	(0.95-1.04)	1.12	(0.76-1.64)	2.16	(0.74-6.33)
Newborn placed on mother's chest or arms	1129	0.998	(0.992-1.004)	3.84	(2.47-5.95)	1.56	(0.95-2.54)	0.89	(0.59-1.32)	1.08	(0.89-1.31)	0.96	(0.90-1.03)	1.06	(0.67-1.67)	3.03	(1.23-7.49)
Cord cut after placenta delivered	424	1.008	(1.002-1.014)	0.1	(0.01-1.13)	1.17	(0.73-1.85)	0.95	(0.63-1.43)	1.06	(0.88-1.27)	1	(0.95-1.06)	0.8	(0.51-1.25)	0.19	(0.05-0.71)
Cord cut with new blade	299	1.011	(1.002-1.019)	0.26	(0.03-2.20)	0.65	(0.36-1.20)	1.49	(0.87-2.55)	0.83	(0.64-1.07)	1.05	(0.95-1.15)	0.78	(0.45-1.35)	1.25	(0.46-3.46)
Anything applied to cord	59	1.007	(0.987-1.027)	1.15	(0.30-4.39)	1.17	(0.23-5.90)	2.14	(0.48-9.48)	1.01	(0.61-1.68)	1	(0.83-1.20)	0.54	(0.12-2.42)	1	-
CHX applied to cord stump	133	1.012	(0.997-1.027)	1.31	(0.52-3.32)	0.2	(0.07-0.57)	1.04	(0.43-2.53)	0.96	(0.70-1.32)	0.99	(0.88-1.11)	1.66	(0.62-4.45)	0.6	(0.13-2.78)
Breastfeeding in first hour	957	1.001	(0.997-1.006)	2.34	(1.68-3.26)	0.73	(0.48-1.11)	1.2	(0.88-1.63)	0.94	(0.82-1.09)	1.01	(0.96-1.06)	0.87	(0.61-1.26)	1.45	(0.65-3.27)

Supplemental Table 6. Characteristics associated with maternal over-reporting, by site of delivery

Facility Delivery	n	Recall period (in weeks)		Delivery complications (none [ref] vs complications)		Child sex (male [ref] vs female)		Parity		Maternal age (in years)		Maternal education (no education [ref] vs any education)		Maternal ethnicity (Madhesi [ref] vs Pahadi)	
		AOR	95% CI	AOR	95% CI	AOR	95% CI	AOR	95% CI	AOR	95% CI	AOR	95% CI	AOR	95% CI
Clean Birth Kit - Any Item Used	358	1.004	(0.997-1.010)	0.63	(0.39-1.01)	0.91	(0.59-1.41)	1.04	(0.84-1.29)	0.98	(0.92-1.04)	1	(0.64-1.57)	1.03	(0.47-2.28)
Clean Birth Kit - Sheet Used	375	1.004	(0.997-1.010)	0.63	(0.39-1.02)	0.94	(0.61-1.44)	1.04	(0.84-1.28)	0.99	(0.93-1.05)	0.88	(0.56-1.38)	0.78	(0.34-1.78)
Clean Birth Kit - Soap Used	400	1.002	(0.995-1.008)	0.58	(0.37-0.91)	1	(0.67-1.51)	1.15	(0.93-1.41)	0.95	(0.89-1.01)	1	(0.65-1.54)	0.67	(0.30-1.49)
Any injection given	18	0.927	(0.851-1.009)	1	-	3.9E6	-	0.59	(0.20-1.74)	1.51	(0.90-2.52)	8.1E7	-	0.58	(0.00-94.93)
Injectable Oxytocin / Ergo given	1														
Newborn wrapped	217	1.006	(0.997-1.015)	0.76	(0.42-1.38)	0.79	(0.45-1.40)	0.94	(0.70-1.26)	1.02	(0.94-1.12)	1.03	(0.55-1.94)	1.3	(0.32-5.28)
Newborn washed	503	1.003	(0.994-1.011)	0.69	(0.32-1.46)	0.43	(0.22-0.84)	1.13	(0.86-1.49)	1.02	(0.93-1.12)	0.55	(0.27-1.14)	1	-
Newborn wiped	201	1.004	(0.994-1.014)	0.66	(0.34-1.28)	0.48	(0.25-0.91)	0.98	(0.71-1.36)	0.98	(0.89-1.08)	0.94	(0.46-1.91)	1.7	(0.33-8.76)
Newborn placed on mother's chest or arms	371	0.999	(0.991-1.007)	1.5	(0.84-2.65)	0.98	(0.58-1.65)	1.12	(0.86-1.45)	0.98	(0.91-1.06)	1.55	(0.89-2.71)	3.41	(1.17-9.94)
Cord cut after placenta delivered	419	1.008	(1.002-1.014)	1.17	(0.73-1.85)	0.93	(0.62-1.40)	1.05	(0.87-1.26)	1.01	(0.95-1.07)	0.82	(0.52-1.27)	0.15	(0.03-0.65)
Cord cut with new blade	289	1.011	(1.002-1.019)	0.66	(0.36-1.22)	1.53	(0.89-2.63)	0.89	(0.68-1.16)	1.04	(0.94-1.14)	0.81	(0.46-1.40)	1.32	(0.48-3.64)
Anything applied to cord	24	1	(0.964-1.037)	0.95	(0.05-18.24)	25.3	(0.25-2519)	3.44	(0.73-16.15)	0.63	(0.38-1.04)	0.3	(0.02-5.82)	1	-
CHX applied to cord stump	90	1.008	(0.988-1.028)	0.13	(0.03-0.47)	0.46	(0.14-1.51)	0.88	(0.58-1.34)	0.98	(0.84-1.14)	2.15	(0.61-7.65)	0.38	(0.07-2.16)
Breastfeeding in first hour	387	1.005	(0.999-1.011)	0.69	(0.42-1.12)	0.96	(0.62-1.48)	0.89	(0.72-1.09)	1.03	(0.97-1.10)	0.82	(0.51-1.32)	1.61	(0.61-4.28)
Home Delivery															
Clean Birth Kit - Any Item Used	50	1.004	(0.980-1.028)	0.67	(0.10-4.65)	1.23	(0.31-4.86)	0.64	(0.32-1.28)	1.01	(0.78-1.31)	0.08	(0.02-0.43)	1	-
Clean Birth Kit - Sheet Used	148	1.01	(0.999-1.021)	0.79	(0.28-2.24)	2.14	(1.04-4.39)	0.9	(0.67-1.21)	1.07	(0.95-1.20)	0.24	(0.10-0.58)	1.4	(0.08-24.73)
Clean Birth Kit - Soap Used	113	1.003	(0.989-1.016)	0.94	(0.23-3.78)	2.58	(1.08-6.17)	0.87	(0.61-1.25)	1.07	(0.94-1.22)	0.21	(0.07-0.65)	1	-
Any injection given	356	1.009	(0.998-1.020)	1.76	(0.36-8.50)	1.28	(0.59-2.79)	0.83	(0.56-1.21)	1	(0.89-1.12)	0.81	(0.28-2.33)	1	-
Injectable Oxytocin / Ergo given	349														
Newborn wrapped	443	1.001	(0.996-1.007)	1.2	(0.59-2.47)	0.64	(0.44-0.93)	1	(0.84-1.19)	0.98	(0.93-1.04)	0.87	(0.53-1.44)	0.91	(0.26-3.16)
Newborn washed	716	1.01	(0.999-1.021)	3.33	(1.25-8.84)	0.75	(0.33-1.69)	0.81	(0.55-1.20)	1.07	(0.95-1.20)	2.27	(0.93-5.56)	1	-
Newborn wiped	493	1.005	(0.999-1.010)	0.8	(0.39-1.66)	0.64	(0.44-0.92)	0.96	(0.81-1.12)	1	(0.95-1.06)	1.23	(0.77-1.95)	2.59	(0.64-10.42)
Newborn placed on mother's chest or arms	758	0.996	(0.986-1.005)	2.11	(0.83-5.35)	0.77	(0.40-1.47)	1.05	(0.78-1.42)	0.93	(0.83-1.03)	0.42	(0.16-1.13)	2.32	(0.28-19.50)
Cord cut after placenta delivered	5														
Cord cut with new blade	10														
Anything applied to cord	35	1.008	(0.977-1.040)	1.69	(0.09-33.22)	1.97	(0.23-16.85)	0.75	(0.36-1.57)	1.19	(0.86-1.64)	0.34	(0.02-4.88)	1	-
CHX applied to cord stump	42	1.009	(0.983-1.036)	0.48	(0.05-5.02)	4.97	(0.85-29.12)	0.88	(0.49-1.58)	1.05	(0.84-1.30)	0.48	(0.06-3.67)	1	-
Breastfeeding in first hour	570	0.998	(0.992-1.004)	0.81	(0.37-1.79)	1.52	(0.98-2.36)	1	(0.82-1.22)	0.99	(0.92-1.05)	0.92	(0.52-1.64)	1.08	(0.22-5.22)

Supplemental Table 7. Characteristics associated with maternal under-reporting

Indicator	n	Recall period (in weeks)		Site of delivery (home [ref] vs facility)		Delivery complications (no complication [ref] vs complications)		Child sex (male [ref] vs female)		Parity		Maternal age (in years)		Maternal education (no education [ref] vs any education)		Maternal ethnicity (Madhesi [ref] vs Pahadi)	
		AOR	95% CI	AOR	95% CI	AOR	95% CI	AOR	95% CI	AOR	95% CI	AOR	95% CI	AOR	95% CI	AOR	95% CI
Clean Birth Kit - Any Item Used	1044	1.008	(1.000-1.016)	17.62	(8.03-38.67)	1.63	(0.84-3.14)	0.84	(0.47-1.49)	1.14	(0.87-1.49)	0.95	(0.87-1.04)	1.1	(0.57-2.12)	3.5	(0.88-13.91)
Clean Birth Kit - Sheet Used	929	1.003	(0.997-1.008)	3.03	(2.00-4.58)	1.45	(0.86-2.45)	0.78	(0.52-1.17)	1.06	(0.89-1.26)	1	(0.94-1.06)	1.36	(0.86-2.15)	1.51	(0.50-4.53)
Clean Birth Kit - Soap Used	939	1.004	(1.000-1.009)	1.25	(0.86-1.81)	1.26	(0.79-2.01)	1.01	(0.73-1.40)	1.02	(0.88-1.18)	1	(0.95-1.05)	1.14	(0.77-1.68)	1.94	(0.79-4.77)
Any injection given	1112	0.999	(0.993-1.004)	0.87	(0.59-1.28)	0.85	(0.53-1.37)	1.36	(0.93-1.97)	1.01	(0.85-1.19)	1.03	(0.98-1.08)	0.73	(0.47-1.14)	0.67	(0.20-2.27)
Injectable Oxytocin / Ergo given	1																
Newborn wrapped	671	1.003	(0.999-1.008)	0.38	(0.27-0.53)	1.24	(0.79-1.95)	0.94	(0.68-1.32)	1.09	(0.93-1.26)	0.99	(0.95-1.04)	0.8	(0.55-1.17)	1.56	(0.65-3.71)
Newborn washed	551	1.003	(0.998-1.009)	0.59	(0.40-0.87)	1.33	(0.83-2.13)	0.91	(0.63-1.32)	1.18	(0.99-1.40)	0.99	(0.94-1.05)	1.07	(0.70-1.64)	0.45	(0.13-1.58)
Newborn wiped	306	1.009	(1.002-1.016)	0.63	(0.21-1.85)	1.03	(0.57-1.85)	0.8	(0.49-1.31)	1.2	(0.95-1.52)	0.96	(0.89-1.02)	1.02	(0.60-1.75)	0.44	(0.18-1.10)
Newborn placed on mother’s chest or arms	873	1.001	(0.993-1.009)	30.83	(17.38-54.71)	0.45	(0.19-1.06)	1.03	(0.59-1.77)	0.78	(0.57-1.05)	0.98	(0.89-1.07)	0.74	(0.39-1.40)	1.53	(0.34-6.95)
Cord cut after placenta delivered	158	1.008	(0.992-1.025)	1	-	0.97	(0.22-4.20)	0.61	(0.18-2.13)	2.34	(1.11-4.91)	0.69	(0.51-0.94)	1.2	(0.31-4.67)	1	-
Cord cut with new blade	1284	1.001	(0.994-1.008)	8.32	(4.45-15.54)	1.51	(0.88-2.59)	0.8	(0.49-1.28)	1.07	(0.86-1.34)	0.96	(0.90-1.04)	0.74	(0.43-1.25)	0.65	(0.15-2.87)
Anything applied to cord	1206	1	(0.992-1.007)	6.92	(3.78-12.67)	1.13	(0.62-2.06)	0.85	(0.52-1.39)	0.97	(0.76-1.24)	1	(0.92-1.08)	0.88	(0.51-1.53)	0.32	(0.04-2.50)
CHX applied to cord stump	530	1.006	(1.000-1.011)	0.41	(0.28-0.60)	1.5	(0.92-2.44)	1.43	(0.99-2.06)	0.94	(0.79-1.11)	1.02	(0.97-1.07)	1.04	(0.69-1.56)	0.27	(0.10-0.68)
Breastfeeding in first hour	1044	1.008	(1.000-1.016)	17.62	(8.03-38.67)	1.63	(0.84-3.14)	0.84	(0.47-1.49)	1.14	(0.87-1.49)	0.95	(0.87-1.04)	1.1	(0.57-2.12)	3.5	(0.88-13.91)

Supplemental Table 8. Characteristics associated with maternal under-reporting, by site of delivery

Facility Delivery	n	Recall period (in weeks)		Delivery complications (none [ref] vs complications)		Child sex (male [ref] vs female)		Parity		Maternal age (in years)		Maternal education (no education [ref] vs any education)		Maternal ethnicity (Madhesi [ref] vs Pahadi)	
		AOR	95% CI	AOR	95% CI	AOR	95% CI	AOR	95% CI	AOR	95% CI	AOR	95% CI	AOR	95% CI
Clean Birth Kit - Any Item Used	291	1.008	(1.000-1.017)	1.78	(0.89-3.55)	0.78	(0.41-1.48)	1.01	(0.74-1.36)	0.99	(0.90-1.09)	1.03	(0.50-2.09)	4.14	(0.88-19.59)
Clean Birth Kit - Sheet Used	274	1.009	(1.001-1.017)	1.8	(0.94-3.41)	0.88	(0.49-1.58)	0.96	(0.73-1.25)	1.01	(0.93-1.10)	1.24	(0.65-2.37)	4	(0.83-19.16)
Clean Birth Kit - Soap Used	249	1.008	(1.000-1.017)	1.45	(0.72-2.92)	1.01	(0.54-1.88)	1.13	(0.84-1.52)	0.94	(0.85-1.04)	1.17	(0.59-2.32)	8.2	(1.38-48.65)
Any injection given	671	0.996	(0.989-1.004)	0.6	(0.33-1.11)	1.25	(0.76-2.07)	0.89	(0.70-1.11)	1.06	(0.99-1.13)	0.81	(0.47-1.40)	0.24	(0.03-1.80)
Injectable Oxytocin / Ergo given	1														
Newborn wrapped	372	1.005	(0.999-1.012)	1.11	(0.64-1.95)	0.97	(0.60-1.57)	0.92	(0.73-1.16)	1.02	(0.95-1.09)	0.82	(0.49-1.36)	1.43	(0.54-3.76)
Newborn washed	2														
Newborn wiped	339	1.008	(1.001-1.015)	1.44	(0.82-2.53)	0.86	(0.52-1.41)	1.01	(0.81-1.25)	1.03	(0.96-1.10)	1.17	(0.69-1.99)	0.52	(0.14-1.91)
Newborn placed on mother's chest or arms	287	1.008	(1.000-1.015)	1.11	(0.61-2.00)	0.81	(0.49-1.34)	1.22	(0.96-1.55)	0.96	(0.89-1.03)	1.12	(0.65-1.93)	0.53	(0.21-1.36)
Cord cut after placenta delivered	118	0.992	(0.981-1.003)	0.43	(0.16-1.15)	1.23	(0.57-2.65)	0.83	(0.53-1.29)	0.97	(0.85-1.11)	1.03	(0.44-2.41)	4.05	(0.41-40.10)
Cord cut with new blade	158	1.008	(0.992-1.025)	0.97	(0.22-4.20)	0.61	(0.18-2.13)	2.34	(1.11-4.91)	0.69	(0.51-0.94)	1.2	(0.31-4.67)	-	
Anything applied to cord	532	1.002	(0.994-1.009)	1.67	(0.95-2.92)	0.85	(0.50-1.43)	1.04	(0.82-1.33)	0.98	(0.90-1.06)	0.66	(0.37-1.17)	0.75	(0.17-3.37)
CHX applied to cord stump	463	1.001	(0.993-1.010)	1.26	(0.67-2.34)	0.72	(0.41-1.28)	0.94	(0.70-1.25)	1	(0.92-1.09)	0.79	(0.43-1.45)	0.37	(0.05-2.89)
Breastfeeding in first hour	303	1.001	(0.994-1.008)	1.54	(0.91-2.62)	1.35	(0.85-2.17)	0.93	(0.74-1.17)	1.01	(0.94-1.08)	1.05	(0.64-1.74)	0.31	(0.11-0.91)
Home Delivery															
Clean Birth Kit - Any Item Used	673	1.005	(0.986-1.024)	1	-	1.33	(0.32-5.51)	2.01	(1.13-3.58)	0.77	(0.60-1.00)	1.39	(0.25-7.58)	1	-
Clean Birth Kit - Sheet Used	640	0.996	(0.988-1.004)	0.45	(0.11-1.91)	0.76	(0.43-1.35)	1.15	(0.91-1.47)	0.98	(0.91-1.07)	1.5	(0.78-2.89)	1	-
Clean Birth Kit - Soap Used	690	1.002	(0.997-1.007)	1	-	1.05	(0.71-1.55)	0.99	(0.83-1.19)	1.01	(0.96-1.07)	1.11	(0.69-1.80)	0.9	(0.25-3.27)
Any injection given	441	1.002	(0.994-1.010)	1.52	(0.70-3.28)	1.4	(0.79-2.49)	1.18	(0.91-1.52)	0.99	(0.91-1.08)	0.53	(0.23-1.23)	2.97	(0.57-15.49)
Injectable Oxytocin / Ergo given	0														
Newborn wrapped	299	1.001	(0.995-1.008)	1.47	(0.67-3.23)	0.93	(0.58-1.48)	1.26	(1.01-1.56)	0.97	(0.91-1.04)	0.73	(0.41-1.30)	1.61	(0.22-12.04)
Newborn washed	1														
Newborn wiped	209	0.998	(0.990-1.006)	1	(0.41-2.46)	1.02	(0.57-1.80)	1.57	(1.16-2.12)	0.93	(0.85-1.02)	0.89	(0.42-1.88)	1	-
Newborn placed on mother's chest or arms	17														
Cord cut after placenta delivered	741	1.01	(0.999-1.021)	0.8	(0.18-3.56)	0.86	(0.38-1.91)	0.74	(0.48-1.14)	0.97	(0.86-1.11)	0.37	(0.11-1.27)	1	-
Cord cut with new blade	785														
Anything applied to cord	677	0.997	(0.981-1.014)	1	-	0.59	(0.18-1.93)	1.22	(0.71-2.08)	0.89	(0.74-1.08)	1.41	(0.41-4.87)	1	-
CHX applied to cord stump	669	0.994	(0.978-1.009)	1	-	1.53	(0.55-4.29)	1.11	(0.70-1.75)	0.99	(0.85-1.15)	1.46	(0.44-4.92)	1	-
Breastfeeding in first hour	227	1.013	(1.004-1.023)	1.32	(0.34-5.06)	1.63	(0.88-3.03)	0.94	(0.72-1.23)	1.04	(0.95-1.13)	0.87	(0.43-1.78)	0.18	(0.03-1.19)
